# Interactive effects of zinc and copper sources and phytase on growth performance, mineral digestibility, bone mineral concentrations, oxidative status, and gut morphology in nursery pigs

**DOI:** 10.1093/tas/txaa083

**Published:** 2020-06-20

**Authors:** Ping Ren, Juxing Chen, Karen Wedekind, Deana Hancock, Mercedes Vázquez-Añón

**Affiliations:** Novus International, Inc., St. Charles, MO

**Keywords:** copper, growth performance, gut morphology, nursery pigs, phytase, zinc

## Abstract

This study investigated the interactive effects of zinc (Zn) and copper (Cu) sources and phytase on growth performance, oxidative status, mineral digestibility, tissue mineral concentrations, and gut morphology in nursery pigs. A total of 288 weaning barrows [body weight (BW) = 5.71 ± 0.81 kg], blocked by initial BW, were randomly allotted to one of eight dietary treatments, with nine pens per treatment and four pigs per pen. The eight dietary treatments were arranged in 2 × 2 × 2 factorial design, with two Zn sources [2,000, 2,000, and 100 mg/kg Zn from zinc oxide (ZnO) during phase 1 (days 1–14) and phase 2 (days 15–28), and phase 3 (days 29–42), respectively; 100 mg/kg Zn from zinc methionine hydroxy analogue chelate (Zn-MHAC) from phases 1 to 3], two Cu sources [150, 80, and 80 mg/kg Cu from copper sulfate (CuSO_4_) or copper methionine hydroxy analogue chelate (Cu-MHAC) during phases 1–3, respectively], and two phytase inclusion levels (0 or 500 FTU/kg). Results showed that ZnO supplementation at 2,000 mg/kg Zn significantly increased average daily feed intake (ADFI; *P* = 0.01) and average daily gain (ADG; *P* = 0.03) during phase 1 compared to Zn-MHAC group; however, Zn-MHAC supplementation tended (*P* = 0.06) to improve gain to feed ratio (G:F) during phase 2 compared to ZnO group. There were no differences (*P* > 0.10) between ZnO and Zn-MHAC groups in terms of ADG, ADFI, and G:F during the entire nursery period. Compared with CuSO_4_, Cu-MHAC tended to increase ADG (*P* = 0.07) and G:F (*P* = 0.08) during the entire nursery period. Phytase supplementation significantly increased ADG (*P* < 0.01), ADFI (*P* < 0.01), and G:F (*P* < 0.01) during the entire nursery period compared with no phytase supplementation. There was a significant interaction (*P* < 0.01) between Zn source and phytase on standardized total tract digestibility (STTD) of phosphorus (P), whereas there was no interaction (*P* = 0.21) between Cu sources and phytase on STTD of P. However, there was a significant interaction between Cu sources and phytase on calcium (Ca; *P* = 0.02) and P (*P* = 0.03) concentrations in metacarpal bones and G:F in phase 2 (*P* = 0.09). Furthermore, pigs fed diets containing Zn-MHAC tended to have lower ileum villus width (*P* = 0.07), compared with those fed diets containing ZnO, and pigs fed diets containing Cu-MHAC tended to have lower plasma malondialdehyde concentration (*P* = 0.10) compared with those fed diets containing CuSO_4_. In conclusion, under the conditions of the current study, ZnO supplementation at 2,000 mg/kg Zn was only effective in the first 2 wk postweaning, whereas Zn-MHAC supplementation at 100 mg/kg Zn could achieve better feed efficiency during phase 2 compared to pharmacological levels of ZnO, therefore, leading to no difference of growth performance in the entire nursery period. Low levels of Zn-MHAC may improve phytase efficacy on degrading phytate P compared to pharmacological levels of ZnO. Cu-MHAC may be more effective to promote growth compared to CuSO_4_, which may be partially driven by reduced oxidative stress. Results also indicated that Cu-MHAC might exert a synergistic effect with phytase on improving feed efficiency and bone mineralization.

## INTRODUCTION

Pharmacological levels of zinc oxide (ZnO) are widely used in the weaning pig diets to promote growth and prevent postweaning diarrhea (Hill et al., 2000; Case and Carlson, 2002; Ou et al., 2007). This practice is not environmentally sustainable; therefore, an effective alternative is warranted to be investigated. Alternatively, a chelated Zn source has been proven to be more bioavailable in both chickens and pigs (Wedekind et al., 1992, 1994) in comparison with ZnO. Indeed, Buff et al. (2005) observed no difference between pigs fed 300 or 450 mg/kg Zn as Zn-polysaccharide and 2,000 mg/kg Zn as ZnO in terms of overall growth performance during 35-d postweaning. It is not known if supplementation of chelated zinc at 100 mg/kg Zn could achieve similar performance as ZnO in nursery pigs. Therefore, the first objective of this study was to evaluate the supplementation of Zn as zinc methionine hydroxy analogue chelate (Zn-MHAC, MINTREX Zn, Novus International Inc., St. Charles, MO) at 100 mg/kg Zn on growth performance, mineral digestibility, tissue mineral concentrations, oxidative status, and gut morphology in nursery pigs in comparison with pharmacological levels of ZnO.

Although the NRC (2012) recommendation for copper (Cu) is 5–6 mg/kg for 5–25 kg nursery pigs, a high level of copper sulfate (CuSO_4_, 150–250 mg/kg Cu) is widely used in weaning pigs to promote growth and improve feed efficiency (Cromwell et al., 1989). Similar to ZnO, high Cu supplementation presents an environmental concern and leads to increased Cu excretion in the feces (Roof and Mahan, 1982). Alternatively, chelated Cu is more bioavailable in both chickens and pigs (Yi et al., 2007; Richards et al., 2010). Studies have demonstrated that supplementation of Cu as Cu citrate (125 mg/kg Cu) or Cu proteinate (100 mg/kg Cu) could achieve similar growth performance in nursery pigs as CuSO_4_ supplementation at 250 mg/kg Cu (Armstrong et al., 2004; Veum et al., 2004). Similarly, supplementation of Cu as copper methionine hydroxy analogue chelate (Cu-MHAC, MINTREX Cu, Novus International Inc., St. Charles, MO) at 150 or 170 mg/kg Cu yielded greater average daily gain (ADG) in the nursery pigs compared with CuSO_4_ at the same Cu inclusion level (Zhao et al., 2014; Ma et al., 2015). However, it is not certain that supplementation of Cu-MHAC at a lower inclusion level could achieve similar performance as CuSO_4_. Therefore, the second objective of this study was to investigate the effect of two Cu sources (Cu-MHAC vs CuSO_4_) at equivalent but decreasing Cu levels during three different phases in the nursery period (150, 80, and 80 mg/kg in phase 1, 2, and 3, respectively) on growth performance, mineral digestibility, tissue mineral concentrations, oxidative status, and gut morphology in nursery pigs.

Compared with chelated Zn and Cu sources, ZnO and CuSO_4_ are easily dissociated in the acidic pH in the stomach, resulting in the formation of Zn-phytate and Cu-phytate complexes (Dintzis et al., 1995; Pang and Applegate, 2006). These complexes could impair phytase efficacy, resulting in lower phosphorus (P) release from the phytate molecule (Banks et al., 2004; Pang and Applegate, 2006; Blavi et al., 2017). An in vitro model has demonstrated that Cu lysine is less inhibitive to phytase compared with CuSO_4_ (Pang and Applegate, 2006). However, it is not known whether chelated trace minerals could improve phytase efficacy in vivo compared with their inorganic counterparts. Therefore, the third objective was to determine the interaction between Zn sources and phytase, and interaction between Cu sources and phytase on growth performance, mineral digestibility, tissue mineral concentrations, oxidative status, and gut morphology in nursery pigs.

## MATERIALS AND METHODS

The animal protocols used in the current study were reviewed and approved by Novus International, Inc. Animal Ethics Committee.

### Animals and Management

The present experiment was conducted at Green Acres Animal Research and Testing Facility (a Novus International, Inc. facility Montgomery City, MO). A total of 288 TR4 × C22 weaning barrows [body weight (BW) = 5.71 ± 0.81 kg; PIC, Hendersonville, TN] were used in this study. Pigs were housed in plastic coated floor pens. Each piglet was tagged for individual identification. Pigs had free access to the feed and water during the entire nursery period. A three-phase feeding program (days 0–14, 15–28, and 29–42) was used in the present study.

### Experimental Design and Dietary Treatments

At the initiation of this study (day 0), piglets were weighed individually and allotted to one of eight dietary treatments according to a randomized complete block design, which was blocked by initial BW. There were nine pens per treatment and four pigs per pen. A basal diet for each phase was formulated to meet the energy and nutrient requirements for different stages of pigs according to the recommendation by NRC (2012), with the exception that standardized total tract digestible (STTD) P was reduced by 0.15% and Ca level was adjusted to meet the fixed ratio of Ca to STTD P of 2.15. The eight dietary treatments were arranged in 2 × 2 × 2 factorial design (Table 1), with two Zn sources [2,000 mg/kg Zn from ZnO during phase 1 (days 1–14) and phase 2 (days 15–28), and 100 mg/kg Zn from ZnO during phase 3 (days 29–42); 100 mg/kg Zn from Zn-MHAC during phases 1–3], two Cu sources [150 mg/kg Cu from CuSO_4_ or Cu-MHAC during phase 1, and 80 mg/kg Cu from CuSO_4_ or Cu-MHAC during phases 2 and 3], and two phytase inclusion levels (0, 500 unit of phytase (FTU)/kg; Quantum Blue, AB Vista, Marlborough, UK). The basal diet composition for the three phases was presented in Table 2.

**Table 1.** Description of dietary treatments in terms of Zn and Cu sources and levels and phytase inclusion levels

Trt	Zn sources	Zn levels in phase 1, 2, and 3 diets, mg/kg	Cu sources	Cu levels in phase 1, 2, and 3 diets, mg/kg	Phytase^*c*^, FTU/kg
1	ZnO	2,000, 2,000, 100	CuSO_4_	150, 80, 80	0
2	ZnO	2,000, 2,000, 100	Cu-MHAC^*b*^	150, 80, 80	0
3	Zn-MHAC^*a*^	100, 100, 100	CuSO_4_	150, 80, 80	0
4	Zn-MHAC	100, 100, 100	Cu-MHAC	150, 80, 80	0
5	ZnO	2,000, 2,000, 100	CuSO_4_	150, 80, 80	500
6	ZnO	2,000, 2,000, 100	Cu-MHAC	150, 80, 80	500
7	Zn-MHAC	100, 100, 100	CuSO_4_	150, 80, 80	500
8	Zn-MHAC	100, 100, 100	Cu-MHAC	150, 80, 80	500

^*a*^Zn-MHAC represents Zn methionine hydroxy analogue chelate (MINTREX Zn), which is manufactured by Novus International Inc., St. Charles, MO.

^*b*^Cu-MHAC represents Cu methionine hydroxy analogue chelate (MINTREX Cu), which is manufactured by Novus International Inc., St. Charles, MO.

^*c*^Phytase used in this study is a commercial feed-grade phytase (Quantum Blue, AB Vista, Marlborough, UK), with analyzed activity of 6,033 FTU/g.

**Table 2.** Ingredient and nutrient composition of basal experimental diets (as-fed basis)

Items	Phase 1 (days 0–14)	Phase 2 (days 15–28)	Phase 3 (days 29–42)
Ingredients, %			
Corn, yellow dent	54.77	57.82	58.54
Canola meal	7.00	5.00	8.00
Soybean meal, 47.5% CP	18.00	20.00	26.00
Defatted rice bran	3.00	3.00	3.00
Plasma spray dried	4.00	1.50	0.00
Whey powder	5.00	5.00	0.00
Fish meal, menhaden	3.00	2.00	0.00
Choice white grease	2.65	2.80	2.60
Limestone	1.00	0.82	0.64
Salt	0.30	0.30	0.30
Vitamin premix^*a*^	0.11	0.11	0.11
Mineral premix^*b*^	0.40	0.40	0.40
Phytase premix^*c*^	0.20	0.20	0.20
Titanium dioxide	0.00	0.40	0.00
L-lysine HCl	0.38	0.43	0.18
MHA^*d*^	0.10	0.10	0.03
L-threonine	0.09	0.12	0.00
Total	100.00	100.00	100.00
Calculated nutrient composition			
ME, kcal/kg	3,401	3,404	3,353
CP, %	22.31	20.29	20.90
SID Lys, %	1.40	1.28	1.07
SID Thr, %	0.82	0.76	0.64
SID Met + Cys, %	0.67	0.59	0.60
SID Trp, %	0.24	0.21	0.21
STTD P, %	0.30	0.25	0.18
Phytate P, %	0.30	0.29	0.34
Analyzed nutrient composition			
Ca, %	0.66	0.67	0.50
Total P, %	0.58	0.55	0.50

CP, crude protein; ME, metabolizable energy; SID, standardized ileal digestible.

^*a*^Supplied the following nutrients per kilogram of diets: vitamin A, 11,000 IU; vitamin D, 1,760 IU; vitamin E, 83.6 IU; vitamin K, 5.5 mg; thiamine, 3.52 mg; riboflavin, 13.2 mg; niacin, 70.4 mg; pantothenic acid, 39.6 mg; pyridoxine, 7.04 mg; folic acid, 1,045 µg; biotin, 275 µg; vitamin B12, 55 µg.

^*b*^Four different mineral premixes are made for each phase according to the treatment description in Table 1. ZnO is included in the respective treatments directly at the expense of corn.

^*c*^Phytase premix is made to provide 500 FTU/kg for the four respective treatments at the inclusion level of 0.2%.

^*d*^MHA is dry calcium salt of D, L-2-hydroxy-4-(methylthio)butanoic acid (84% Met activity, MHA, Novus International, Inc., St. Charles, MO).

### Measurements and Sample Collection

Piglet BW were measured at the initiation of the study (day 0) and at the end of each phase (days 14, 28 and 42). Feed addition to each feeder was recorded each time when the feed was added to the feeder. At the end of each phase, remaining feed in the feeder was weighed. Average daily gain, average daily feed intake (ADFI), and gain to feed ratio (G:F) were calculated for each phase and the entire nursery period.

Fresh fecal samples were collected via grab sampling from each pig in each pen twice a day from day 24 to 26. The fecal samples were stored at −20 °C immediately after collection. At the end of sample collection, fecal samples within each pen were thawed, homogenized, and immediately placed in a heated oven (NHP-PD-ECO, Win-Holt, Woodbury, NY) at 65 °C for 48 h. All dried fecal samples were ground using a rotor mill (Pulverisette 14, Fritsch GmbH, Idar-Oberstein, Germany) fitted with a 1-mm screen. Ground feces were thoroughly homogenized and a subsample was collected for chemical analysis.

At the end of the study (day 42), one pig from each pen with the BW closest to the average BW of the pen was selected and approximately 7 mL blood samples were collected via jugular puncture, followed by euthanasia using captive bolt. The blood samples were centrifuged at 2,500 rpm at 4 °C for 15 min to obtain plasma samples. Two-centimeter segments of duodenum (10 cm distal to pylorus), mid-jejunum, and ileum (5 cm proximal to the end of small intestine) were collected. These samples were flushed and fixed in the NOTOXhisto fixative (Scientific Device Laboratory, Des Plaines, IL) for morphometry examination and measurements. The third metacarpal samples were collected from selected pigs. The metacarpal samples were stored at −20 °C until chemical analysis.

### Chemical and Biological Analyses

Diets samples were analyzed for dry matter (DM; method 934.01; AOAC, 2006), calcium (Ca; method 985.01; AOAC, 2006), P (method 985.01; AOAC, 2006), Zn (method 985.01; AOAC, 2006), and Cu (method 985.01; AOAC, 2006). Phytase activity in the diets was analyzed according to ISO (2009). Fecal samples were analyzed in duplicate for DM (method 934.01; AOAC, 2006), Ca (method 985.01; AOAC, 2006), and P (method 985.01; AOAC, 2006). Titanium concentration in phase 2 diets and fecal samples was analyzed according to the procedures described by Myers et al. (2004).

Plasma samples were used for malondialdehyde (MDA) analysis, which was quantified using thiobarbituric acid reactive substances assay kit 10009055 (Cayman Chemical Company, Ann Arbor, MI).

Metacarpal samples were ashed in a muffle furnace at 600 °C overnight in porcelain crucibles and the metacarpal ash percentage was expressed as grams of ash per 100 g of dry, fat-free metacarpal weight. The concentrations of Ca, P, Zn, and Cu in the tibia were determined using the procedure described by method 985.01 (AOAC, 2006).

Fixed duodenum, jejunum, and ileum samples were processed, embedded, and sectioned to 5-μm sections and stained with hematoxylin and eosin. The duodenum, jejunum, and ileum morphometry was measured under an Olympus light microscope using Olympus MircroSuite Imaging software (Center Valley, PA). Ten representative villi and crypts were selected to measure villus height, villus width, and crypt depth, and the average values were used in statistical analysis. Villi height was measured from the top of the villi to the top of the lamina propria, whereas villi width was measured at the middle of each villus. Crypt depth was measured from the base upward to the region of transition between the crypt and villi.

### Calculations

The apparent total tract digestibility (ATTD) coefficient for Ca and P in each treatment was calculated according to equations proposed by NRC (2012). STTD of Ca and P were calculated by accounting for endogenous losses of Ca (330 mg/kg DMI; Merriman and Stein, 2016) and P (190 mg/kg DMI; NRC, 2012), respectively.

### Statistical Analysis

SAS 9.4 (SAS Inst. Inc., Gary, NC) was used for all data analysis. Pen served as the experimental unit. The LSMEANS statement was used to calculate the least square means. Tukey–Kramer adjustment was used for multiple comparisons of the least square means. Pooled SEM was calculated for each measurement. A probability of *P* ≤ 0.05 was considered as significant and 0.05 < *P* ≤ 0.10 was declared as a trend.

The GLIMMIX procedure was used to analyze all the data. Zn source, Cu source, phytase, and their interactions were considered as the fixed effects, whereas block was considered as the random effect. All the data were analyzed using default normal regression model. Three-way interactions were not significant for any of the parameters; therefore, they were excluded from the final model.

## RESULTS

### Effect of Zn Sources on Growth Performance

Pigs fed diets containing 2,000 mg/kg Zn from ZnO had significantly higher ADG (194.62 vs. 178.94 g/d; *P* = 0.03; Tables 3 and 4), ADFI (286.09 vs. 268.80 g/d; *P* = 0.01) in phase 1, and BW (8.44 vs. 8.22 kg; *P* = 0.03) on day 14 compared with pigs fed diets containing 100 mg/kg Zn from Zn-MHAC. However, pigs fed diets containing 100 mg/kg Zn from Zn-MHAC tended to have higher G:F (0.80 vs. 0.77 g/g; *P* = 0.06) in phase 2 compared with pigs fed diets containing 2,000 mg/kg Zn from ZnO. There were no differences (*P* > 0.10) among the two Zn sources in terms of ADG, ADFI, and G:F during phase 3 and the entire nursery period, as well as the BW on days 28 and 42.

**Table 3.** Least square means of interactive effects of Zn and Cu sources and phytase on growth performance in nursery pigs

	Main effects						Interactions							
							ZnO				Zn-MHAC^*b*^			
	Zn sources		Cu sources		Phytase^*a*^		CuSO_4_		Cu-MHAC^*c*^		CuSO_4_		Cu-MHAC	
Items	ZnO	Zn-MHAC	CuSO_4_	Cu-MHAC	No	Yes	No	Yes	No	Yes	No	Yes	No	Yes
BW, kg														
Days 0	5.7	5.7	5.7	5.7	5.7	5.7	5.7	5.7	5.7	5.7	5.7	5.8	5.7	5.7
Days 14	8.4	8.2	8.3	8.4	8.2	8.5	8.3	8.6	8.4	8.5	8.0	8.2	8.2	8.5
Days 28	15.2	15.1	15.1	15.3	14.6	15.8	14.6	15.9	14.3	16.1	14.7	15.3	14.8	15.9
Days 42	22.3	22.2	21.9	22.6	20.7	23.9	20.3	23.7	20.8	24.5	20.4	23.4	21.2	24.0
ADG, g/d														
Days 0–14	195	179	183	191	178	196	185	206	188	199	163	178	175	200
Days 14–28	484	495	488	491	457	522	451	521	425	539	478	503	474	525
Days 28–42	505	507	487	525	432	579	403	558	455	603	409	577	461	579
Days 0–28	340	337	336	341	317	359	318	363	307	371	321	340	324	363
Days 0–42	400	399	391	408	361	438	350	434	364	453	354	424	375	441
ADFI, g/d														
Days 0–14	286	269	276	279	276	279	281	296	283	283	269	257	272	277
Days 14–28^*d*^	628	622	629	621	611	640	605	666	579	662	626	619	632	611
Days 28–42^*e*^	952	970	934	988	894	1,027	825	1,020	910	1,052	867	1,023	974	1,013
Days 0–28	456	445	453	449	442	459	443	481	429	472	448	438	450	444
Days 0–42	614	615	609	620	587	643	568	656	577	658	584	629	618	628
G:F, g/g														
Days 0–14	0.68	0.66	0.66	0.68	0.64	0.70	0.65	0.69	0.66	0.70	0.60	0.68	0.64	0.72
Days 14–28^*f*^	0.77	0.80	0.78	0.80	0.76	0.82	0.75	0.78	0.74	0.82	0.77	0.81	0.76	0.87
Days 28–42	0.53	0.52	0.52	0.53	0.48	0.56	0.49	0.55	0.50	0.58	0.47	0.56	0.47	0.57
Days 0–28	0.75	0.76	0.74	0.76	0.72	0.78	0.72	0.75	0.72	0.79	0.72	0.77	0.73	0.82
Days 0–42	0.65	0.65	0.64	0.66	0.62	0.68	0.62	0.66	0.63	0.69	0.60	0.67	0.61	0.70

^*a*^No and yes represent 0 and 500 FTU/kg phytase in the diets, respectively.

^*b*^Zn-MHAC represents Zn methionine hydroxy analogue chelate (MINTREX Zn), which is manufactured by Novus International Inc., St. Charles, MO.

^*c*^Cu-MHAC represents Cu methionine hydroxy analogue chelate (MINTREX Cu), which is manufactured by Novus International Inc., St. Charles, MO.

^*d*^There was a significant interaction (*P* = 0.03) between Zn sources and phytase. Specifically, when ZnO was used as the Zn source, phytase supplementation increased ADFI during phase 2 by 12.16% (664 vs. 592 g/d) compared with no phytase supplementation, whereas when Zn-MHAC was used as the Zn source, phytase supplementation reduced ADFI during phase 2 by 2.23% (615 vs. 629 g/d) compared with no phytase supplementation.

^*e*^There tended to be an interaction (*P* = 0.06) between Cu sources and phytase. Specifically, when CuSO_4_ was used as the Cu source, phytase supplementation increased ADFI during phase 3 by 20.69% (1,021 vs. 846 g/d) compared with no phytase supplementation, whereas when Cu-MHAC was used as the Cu source, phytase supplementation increased ADFI during phase 3 by 9.66% (1,033 vs. 942 g/d) compared with no phytase supplementation.

^*f*^There tended to be an interaction (*P* = 0.09) between Cu sources and phytase. Specifically, when Cu sulfate was used as the Cu source, phytase supplementation significantly increased G:F during phase 2 by 5.26% (0.80 vs. 0.76 g/g) compared with the group with no phytase supplementation, whereas when Cu-MHAC was used as the Cu source, phytase supplementation significantly increased G:F during phase 2 by 12% (0.84 vs. 0.75 g/g) compared with the group with no phytase supplementation.

**Table 4.** SEM and probability values of interactive effects of Zn and Cuc sources and phytase on growth performance in nursery pigs

		*P* values					
Items	Pooled SEM	Zn	Cu	Phytase	Zn × Cu	Zn × phytase	Cu × phytase
BW, kg							
Days 0	0.01	0.86	0.82	0.17	0.45	0.20	0.25
Days 14	0.1	0.03	0.31	0.01	0.23	0.68	0.88
Days 28	0.2	0.79	0.60	<0.01	0.49	0.23	0.40
Days 42	0.3	0.85	0.08	<0.01	0.95	0.35	0.96
ADG, g/d							
Days 0–14	5	0.03	0.29	0.01	0.19	0.81	0.98
Days 14–28	12	0.51	0.88	<0.01	0.70	0.12	0.30
Days 28–42	10	0.89	0.01	<0.01	0.44	0.78	0.32
Days 0–28	7	0.80	0.60	<0.01	0.47	0.22	0.38
Days 0–42	7	0.86	0.07	<0.01	0.88	0.33	0.97
ADFI, g/d							
Days 0–14	5	0.01	0.70	0.75	0.23	0.42	0.94
Days 14–28	13	0.74	0.67	0.12	0.71	0.03	0.91
Days 28–42	16	0.43	0.02	<0.01	0.83	0.12	0.06
Days 0–28	8	0.30	0.70	0.13	0.46	0.03	0.85
Days 0–42	8	0.96	0.31	<0.01	0.61	0.01	0.35
G:F, g/g							
Days 0–14	0.01	0.42	0.19	<0.01	0.37	0.25	0.95
Days 14–28	0.01	0.06	0.29	<0.01	0.77	0.67	0.09
Days 28–42	0.01	0.45	0.29	<0.01	0.58	0.27	0.64
Days 0–28	0.01	0.29	0.12	<0.01	0.74	0.55	0.19
Days 0–42	0.01	0.80	0.08	<0.01	0.88	0.17	0.29

### Effect of Cu Sources on Growth Performance

Pigs fed diets containing 80 mg/kg Cu from Cu-MHAC had significantly higher ADG (524.64 vs. 486.61 g/d; *P* = 0.01; Tables 3 and 4) and ADFI (987.53 vs. 933.75 g/d; *P* = 0.02) in phase 3 compared with pigs fed diets containing 80 mg/kg Cu from CuSO_4_. Additionally, pigs fed diets containing Cu-MHAC tended to have greater ADG (408.33 vs. 390.80 g/d; *P* = 0.07) and G:F (0.66 vs. 0.64 g/g; *P* = 0.08) during the entire nursery period, as well as greater BW on day 42 (22.63 vs. 21.92 kg; *P* = 0.08) compared with pigs fed diets containing CuSO_4_.

### Effect of Phytase on Growth Performance

Pigs fed diets supplemented with 500 FTU/kg had significantly higher ADG (*P* < 0.01; Tables 3 and 4) and G:F (*P* < 0.01) during all three phases and the entire nursery period. Additionally, phytase supplementation significantly increased ADFI during phase 3 (1,027.05 vs. 894.22 g/d; *P* < 0.01) and the entire nursery period (642.73 vs. 586.60 g/d; *P* < 0.01).

### Effect of Interactions Among Zn and Cu Sources and Phytase on Growth Performance

There was a significant interaction between Zn sources and phytase in terms of ADFI during phase 2 (*P* = 0.03; Tables 3 and 4). Specifically, when ZnO was used as the Zn source, phytase supplementation increased ADFI during phase 2 by 12.16% (664 vs. 592 g/d) compared with no phytase supplementation, whereas when Zn-MHAC was used as the Zn source, phytase supplementation reduced ADFI during phase 2 by 2.23% (615 vs. 629 g/d) compared with no phytase supplementation.

There tended to be an interaction (*P* = 0.06) between Cu sources and phytase in terms of ADFI during phase 3. Specifically, when CuSO_4_ was used as the Cu source, phytase supplementation increased ADFI during phase 3 by 20.69% (1,021 vs. 846 g/d) compared with no phytase supplementation, whereas, when Cu-MHAC was used as the Cu source, phytase supplementation increased ADFI during phase 3 by 9.66% (1,033 vs. 942 g/d) compared with no phytase supplementation. Additionally, Cu sources and phytase tended (*P* = 0.09) to interact with each other on G:F during phase 2. Specifically, when CuSO_4_ was used as the Cu source, phytase supplementation increased G:F during phase 2 by 5.26% (0.80 vs. 0.76 g/g) compared with the group with no phytase supplementation, whereas, when Cu-MHAC was used as the Cu source, phytase supplementation increased G:F during phase 2 by 12% (0.84 vs. 0.75 g/g) compared with the group with no phytase supplementation.

### Effect of Zn and Cu Sources and Phytase on Plasma MDA Concentration and Gut Morphology

Pigs fed diets containing Zn at 100 mg/kg from Zn-MHAC in the entire nursery period tended to have higher plasma MDA concentration (10.26 vs. 9.35 µM; *P* = 0.10; Tables 5 and 6) compared with their counterparts with ZnO supplementation. Ileal villus width (135.52 vs. 150.82 µm; *P* = 0.07) tended to be smaller in pigs fed diets containing Zn-MHAC at 100 mg/kg Zn in the entire nursery period compared with pigs fed diets containing ZnO at 2,000 mg/kg Zn during phases 1 and 2 and 100 mg/kg Zn during phase 3. Additionally, pigs fed diets containing Cu-MHAC tended to have lower plasma MDA concentration (9.35 vs. 10.27 µM; *P* = 0.10) compared with pigs fed diets containing CuSO_4_.

**Table 5.** Least square means of interactive effects of Zn and Cu sources and phytase on MDA and gut morphology in nursery pigs

	Main effects						Interactions							
							ZnO				Zn-MHAC^*b*^			
	Zn sources		Cu sources		Phytase^*a*^		CuSO_4_		Cu-MHAC^*c*^		CuSO_4_		Cu-MHAC	
Items	ZnO	Zn-MHAC^*a*^	CuSO4	Cu-MHAC^*b*^	No	Yes	No	Yes	No	Yes	No	Yes	No	Yes
MDA^*d*^, µM	9.35	10.26	10.27	9.35	10.52	9.09	10.65	8.76	10.06	7.93	10.84	10.81	10.54	8.86
Duodenum														
Villus height, µm	830.96	813.33	820.37	823.92	804.10	840.19	798.30	852.12	802.14	871.26	823.47	807.58	792.48	829.80
Villus width, µm	178.39	173.59	178.97	173.01	176.36	175.62	179.86	175.58	176.98	181.16	179.76	180.70	168.86	165.06
Crypt depth, µm	104.02	105.79	106.70	103.11	104.47	105.35	110.31	103.96	105.02	96.80	106.79	105.75	95.74	114.88
VH:CD^*e*^	8.15	7.92	7.83	8.25	7.88	8.20	7.31	8.39	7.71	9.21	7.77	7.84	8.73	7.35
VH:VW^*f*^	4.69	4.73	4.63	4.79	4.61	4.81	4.50	4.87	4.57	4.82	4.64	4.49	4.71	5.07
Jejunum														
Villus height, µm	852.55	873.60	852.18	873.97	828.79	897.36	804.34	866.69	791.14	948.04	857.29	880.40	862.39	894.32
Villus width, µm	167.14	165.65	165.51	167.28	162.86	169.94	153.01	169.05	170.08	176.43	166.68	173.32	161.66	160.94
Crypt depth, µm	88.89	88.34	89.11	88.13	85.60	91.64	85.84	95.61	84.51	89.62	86.23	88.74	85.83	92.57
VH:CD	9.75	10.09	9.76	10.08	9.84	9.99	9.45	9.42	9.37	10.75	10.17	9.98	10.38	9.81
VH:VW	5.15	5.31	5.20	5.26	5.13	5.34	5.31	5.16	4.66	5.48	5.18	5.16	5.35	5.55
Ileum														
Villus height, µm	611.62	606.45	603.47	614.61	595.25	622.83	600.90	616.19	617.89	611.49	571.01	625.77	591.19	637.86
Villus width, µm	150.82	135.52	139.90	146.43	140.42	145.92	142.36	140.05	155.97	164.89	139.18	138.01	124.15	140.73
Crypt depth, µm	116.19	121.70	118.83	119.06	121.43	116.46	116.75	118.11	114.53	115.38	125.49	114.97	128.95	117.38
VH:CD	5.44	5.18	5.25	5.37	5.11	5.51	5.28	5.42	5.59	5.48	4.70	5.60	4.88	5.53
VH:VW	4.27	4.57	4.41	4.42	4.35	4.49	4.32	4.52	4.06	4.18	4.19	4.64	4.83	4.62

^*a*^No and yes represent 0 and 500 FTU/kg phytase in the diets, respectively.

^*b*^Zn-MHAC represents Zn methionine hydroxy analogue chelate (MINTREX Zn), which is manufactured by Novus International Inc., St. Charles, MO.

^*c*^Cu-MHAC represents Cu methionine hydroxy analogue chelate (MINTREX Cu), which is manufactured by Novus International Inc., St. Charles, MO.

^*d*^MDA represents plasma malondialdehyde concentration.

^*e*^VH:CD represent villus height to crypt depth ratio.

^*f*^VH:VW represent villus height to villus width ratio.

**Table 6.** SEM and probability values of interactive effects of Zn and Cu sources and phytase on MDA and gut morphology in nursery pigs

		*P* values					
Items	Pooled SEM	Zn	Cu	Phytase	Zn × Cu	Zn × phytase	Cu × phytase
MDA^*a*^, µM	0.42	0.10	0.10	0.03	0.71	0.30	0.40
Duodenum							
Villus height, µm	14.94	0.40	0.87	0.09	0.71	0.23	0.42
Villus width, µm	3.52	0.33	0.23	0.88	0.14	0.89	0.85
Crypt depth, µm	2.57	0.63	0.32	0.81	0.47	0.03	0.21
VH:CD^*b*^	0.27	0.54	0.26	0.40	0.62	0.01	0.49
VH:VW^*c*^	0.10	0.77	0.24	0.14	0.26	0.46	0.47
Jejunum							
Villus height, µm	17.82	0.40	0.39	0.01	0.63	0.11	0.31
Villus width, µm	2.81	0.71	0.66	0.08	0.01	0.30	0.28
Crypt depth, µm	2.50	0.88	0.78	0.09	0.45	0.69	0.98
VH:CD	0.29	0.41	0.43	0.71	0.46	0.20	0.53
VH:VW	0.14	0.44	0.77	0.29	0.26	0.55	0.14
Ileum							
Villus height, µm	10.94	0.74	0.47	0.08	0.75	0.14	0.63
Villus width, µm	5.80	0.07	0.43	0.50	0.12	0.79	0.38
Crypt depth, µm	4.07	0.34	0.97	0.39	0.64	0.29	0.95
VH:CD	0.19	0.32	0.64	0.14	0.80	0.15	0.63
VH:VW	0.14	0.14	0.96	0.48	0.13	0.91	0.36

^*a*^MDA represents plasma malondialdehyde concentration.

^*b*^VH:CD represent villus height to crypt depth ratio.

^*c*^VH:VW represent villus height to villus width ratio.

Phytase supplementation significantly reduced plasma MDA concentration (9.09 vs. 10.52 µm; *P* = 0.03), increased villus height in jejunum (897.36 vs. 828.79 µm; *P* = 0.01), and tended to increase villus height in duodenum (840.19 vs. 804.10 µm; *P* = 0.09) and ileum (622.83 vs. 595.25 µm; *P* = 0.08) and decrease villus width in jejunum (162.86 vs. 169.94 µm, *P* = 0.08) compared with no phytase supplementation.

### Effect of Zn Sources and Interaction With Phytase on Ca and P Digestibility

Zn-MHAC supplementation at 100 ppm Zn significantly increased STTD of Ca (59.46% vs. 53.83%; *P* < 0.01; Tables 7 and 8) compared with ZnO at 2,000 ppm. There was no interaction (*P* = 0.67) between Zn sources and phytase in terms of STTD of Ca. The absolute increase of STTD of Ca for Zn-MHAC compared with ZnO was 4.81% and 6.45% (Table 9) for without and with phytase supplementation, respectively.

**Table 7.** Least square means of interactive effects of Zn and Cu sources and phytase on total tract digestibility of Ca and P in nursery pigs

	Main effects						Interactions							
							ZnO				Zn-MHAC^*b*^			
	Zn sources		Cu source		Phytase^*a*^		CuSO_4_		Cu-MHAC^*c*^		CuSO_4_		Cu-MHAC	
Items	ZnO	Zn-MHAC	CuSO_4_	Cu-MHAC	No	Yes	No	Yes	No	Yes	No	Yes	No	Yes
ATTD, %														
Ca	49.40	55.22	50.58	54.04	46.57	58.04	39.77	56.66	48.69	52.49	46.75	59.15	51.09	63.88
P	15.06	36.50	26.78	24.77	14.63	36.93	−0.92	33.18	−0.01	27.52	29.96	41.58	29.48	44.97
STTD, %														
Ca	53.83	59.46	54.98	58.32	50.89	62.41	44.06	61.37	52.91	56.99	51.04	63.45	55.55	67.81
P	18.17	39.50	29.83	27.84	17.66	40.02	2.16	36.31	3.17	30.59	32.77	44.74	32.52	47.97

^*a*^No and yes represent 0 and 500 FTU/kg phytase in the diets, respectively.

^*b*^Zn-MHAC represents Zn methionine hydroxy analogue chelate (MINTREX Zn), which is manufactured by Novus International Inc., St. Charles, MO.

^*c*^Cu-MHAC represents Cu methionine hydroxy analogue chelate (MINTREX Cu), which is manufactured by Novus International Inc., St. Charles, MO.

**Table 8.** SEM and probability values of interactive effects of Zn and Cu sources and phytase on total tract digestibility of Ca and P in nursery pigs

		*P* values					
Items	Pooled SEM	Zn	Cu	Phytase	Zn × Cu	Zn × phytase	Cu × phytase
ATTD, %							
Ca	1.35	<0.01	0.07	<0.01	0.57	0.56	0.10
P	1.32	<0.01	0.29	<0.01	0.07	<0.01	0.24
STTD, %							
Ca	1.35	<0.01	0.09	<0.01	0.57	0.67	0.08
P	1.32	<0.01	0.29	<0.01	0.07	<0.01	0.21

**Table 9.** Effect of Zn sources and phytase on total tract digestibility of Ca and P in nursery pigs

	No phytase		With phytase^*a*^			*P* values		
Items	ZnO	Zn-MHAC^*b*^	ZnO	Zn-MHAC	SEM	Zn	Phytase	Zn × phytase
ATTD, %								
Ca	44.23	48.92	54.57	61.51	1.90	<0.01	<0.01	0.56
P	−0.46	29.72	30.58	43.27	1.86	<0.01	<0.01	<0.01
STTD, %								
Ca	48.49	53.3	59.18	65.63	2.69	<0.01	<0.01	0.56
P	2.67	32.65	33.68	46.36	2.63	<0.01	<0.01	<0.01

^*a*^With 500 FTU/kg phytase supplementation in the diets.

^*b*^Zn-MHAC represents Zn methionine hydroxy analogue chelate (MINTREX Zn), which is manufactured by Novus International Inc., St. Charles, MO.

Zn-MHAC supplementation at 100 ppm Zn significantly (*P* < 0.01; Table 9) increased STTD of P without phytase supplementation (32.65% vs. 2.67%) or with phytase supplementation (46.36% vs. 33.68%) compared with ZnO at 2,000 ppm. Additionally, there was a significant (*P* < 0.01) interaction between Zn sources and phytase in terms of STTD of P. Specifically, when ZnO was used as Zn source, phytase supplementation led to an absolute increase of STTD of P by 31.01% (33.68% vs. 2.67%), whereas, when Zn-MHAC was used as Zn source, phytase supplementation led to an absolute increase of STTD of P by 13.71% (46.36% vs. 32.65%).

### Effect of Cu Sources and Interaction With Phytase on Ca and P Digestibility

There tended (*P* = 0.08; Table 10) to be interaction between Cu sources and phytase in terms of STTD of Ca. Specifically, when CuSO_4_ was used as the Cu source, phytase supplementation led to an absolute increase of STTD of Ca by 14.86% (62.41% vs. 47.55%), whereas when Cu-MHAC was used as the Cu source, phytase supplementation led to an absolute increase of STTD of Ca by 8.17% (62.40% vs. 54.23%). Additionally, there were no significant differences (*P* = 0.21) between Cu-MHAC and CuSO_4_ in terms of STTD of P.

**Table 10.** Effect of Cu sources and phytase on total tract digestibility of Ca and P in nursery pigs

	No phytase		With phytase^*a*^			*P* values		
Items	CuSO_4_	Cu-MHAC^*b*^	CuSO_4_	Cu-MHAC	SEM	Cu	Phytase	Cu × phytase
ATTD, %								
Ca	43.26	49.89	57.90	58.18	1.90	0.07	<0.01	0.10
P	14.52	14.74	39.04	34.81	1.86	0.29	<0.01	0.24
STTD, %								
Ca	47.55	54.23	62.41	62.40	1.90	0.09	<0.01	0.08
P	17.47	17.84	42.19	37.84	1.86	0.21	<0.01	0.21

^*a*^With 500 FTU/kg phytase supplementation in the diets.

^*b*^Cu-MHAC represents Cu methionine hydroxy analogue chelate (MINTREX Cu), which is manufactured by Novus International Inc., St. Charles, MO.

### Effect of Zn and Cu Sources and Phytase on Metacarpal Mineral Concentrations

There was a significant interaction (*P* = 0.01; Tables 11–13) between Zn sources and phytase in terms of metacarpal Zn concentration. Specifically, when ZnO was used as the Zn source, phytase supplementation increased metacarpal Zn concentration by 16.81% (128.61 vs. 110.10 mg/kg), whereas, when Zn-MHAC was used as the Zn source, phytase supplementation reduced metacarpal Zn concentration by 0.74% (104.44 vs. 105.22 mg/kg).

**Table 11.** Least square means of interactive effects of Zn and Cu sources and phytase on metacarpal mineral concentrations (DM basis) in nursery pigs

	Main effects						Interactions							
							ZnO				Zn-MHAC^*b*^			
	Zn source		Cu source		Phytase^*a*^		CuSO_4_		Cu-MHAC^*c*^		CuSO_4_		Cu-MHAC	
Items	ZnO	Zn-MHAC	CuSO_4_	Cu-MHAC	No	Yes	No	Yes	No	Yes	No	Yes	No	Yes
Zn, mg/kg	119.35	104.83	112.27	111.91	107.66	116.52	111.18	125.45	109.02	131.76	108.09	104.37	102.35	104.51
Cu, mg/kg	1.06	0.96	1.22	0.79	1.60	0.42	2.21	0.57	1.21	0.24	1.62	0.48	1.34	0.38
Ca, %	13.76	13.55	13.59	13.72	12.80	14.51	13.28	14.19	12.69	14.88	12.80	14.08	12.42	14.89
P, %	7.10	7.04	7.01	7.13	6.55	7.59	6.73	7.37	6.51	7.79	6.56	7.38	6.38	7.83
Ash, %	39.04	38.44	38.55	38.93	36.32	41.16	36.97	40.58	36.32	42.27	36.41	40.24	35.58	41.54

^*a*^No and yes represent 0 and 500 FTU/kg phytase in the diets, respectively.

^*b*^Zn-MHAC represents Zn methionine hydroxy analogue chelate (MINTREX Zn), which is manufactured by Novus International Inc., St. Charles, MO.

^*c*^Cu-MHAC represents Cu methionine hydroxy analogue chelate (MINTREX Cu), which is manufactured by Novus International Inc., St. Charles, MO.

**Table 12.** SEM and probability values of interactive effects of Zn and Cu sources and phytase on metacarpal mineral concentrations (DM basis) in nursery pigs

		*P* values					
Items	Pooled SEM	Zn	Cu	Phytase	Zn × Cu	Zn × phytase	Cu × phytase
Zn, mg/kg	2.46	<0.01	0.92	0.01	0.48	0.01	0.30
Cu, mg/kg	0.18	0.70	0.70	<0.01	0.36	0.63	0.42
Ca, %	0.18	0.42	0.62	<0.01	0.75	0.53	0.02
P, %	0.10	0.64	0.41	<0.01	0.90	0.55	0.03
Ash, %	0.49	0.39	0.58	<0.01	0.84	0.93	0.10

**Table 13.** Effect of Zn sources and phytase on metacarpal mineral concentrations (DM basis) in nursery pigs

	No phytase		With phytase^*a*^			*P* values		
Items	ZnO	Zn-MHAC^*b*^	ZnO	Zn-MHAC	SEM	Zn	Phytase	Zn × phytase
Zn, mg/kg	110.10	105.22	128.61	104.44	3.42	<0.01	0.01	0.01
Cu, mg/kg	1.71	1.48	0.41	0.43	0.26	0.70	<0.01	0.63
Ca, %	12.98	12.61	14.54	14.49	0.27	0.42	<0.01	0.53
P, %	6.62	6.47	7.58	7.60	0.14	0.64	<0.01	0.55
Ash, %	36.65	36.00	41.42	40.89	0.71	0.39	<0.01	0.93

^*a*^With 500 FTU/kg phytase supplementation in the diets.

^*b*^Zn-MHAC represents Zn methionine hydroxy analogue chelate (MINTREX Zn), which is manufactured by Novus International Inc., St. Charles, MO.

There were significant interactions between Cu sources and phytase in terms of metacarpal Ca (*P* = 0.02; Tables 11, 12 and 14) and P (*P* = 0.03). Specifically, when CuSO_4_ was used as the Cu source, phytase supplementation increased metacarpal Ca (14.14% vs. 13.04%) and P (7.38% vs. 6.65%) concentrations by 8.44% and 10.98%, respectively, whereas, when Cu-MHAC was used as the Cu source, phytase supplementation increased metacarpal Ca (14.89% vs. 12.55%) and P (7.81% vs. 6.44%) concentrations by 18.65% and 21.27%, respectively. Similarly, there tended to be an interaction between Cu sources and phytase in terms of metacarpal ash (*P* = 0.10). Specifically, when CuSO_4_ was used as the Cu source, phytase supplementation increased metacarpal ash concentration by 10.14% (40.41% vs. 36.69%), whereas, when Cu-MHAC was used as the Cu source, phytase supplementation increased metacarpal ash concentration by 16.58% (41.91% vs. 35.95%).

**Table 14.** Effect of Cu sources and phytase on metacarpal mineral concentrations (DM basis) in nursery pigs

	No phytase		With phytase^*a*^			*P* values		
Items	CuSO_4_	Cu-MHAC^*b*^	CuSO_4_	Cu-MHAC	SEM	Cu	Phytase	Cu × phytase
Zn, mg/kg	109.63	105.68	114.91	118.14	3.54	0.92	0.01	0.30
Cu, mg/kg	1.92	1.27	0.53	0.31	0.26	0.70	<0.01	0.42
Ca, %	13.04	12.55	14.14	14.89	0.26	0.62	<0.01	0.02
P, %	6.65	6.44	7.38	7.81	0.14	0.41	<0.01	0.03
Ash, %	36.69	35.95	40.41	41.91	0.71	0.58	<0.01	0.10

^*a*^With 500 FTU/kg phytase supplementation in the diets.

^*b*^Cu-MHAC represents Cu methionine hydroxy analogue chelate (MINTREX Cu), which is manufactured by Novus International Inc., St. Charles, MO.

Phytase supplementation significantly increased metacarpal Zn (116.52 vs. 107.66 mg/kg; *P* < 0.01; Tables 11 and 12) compared with no phytase supplementation. However, metacarpal Cu concentration (0.42 vs. 1.60 mg/kg; *P* < 0.01) was lower in pigs fed diets containing phytase compared with those fed diets without phytase supplementation.

## DISCUSSION

### Zn Sources on Growth Performance and Gut Morphology

In the past several decades, pharmacological levels of ZnO have been widely used in the swine industry as a nutritional or therapeutic strategy to promote growth and prevent diarrhea in newly weaned piglets. A large body of literature has demonstrated the effectiveness of high levels of ZnO ranging from 2,000 to 3,000 mg/kg of Zn, which could increase ADG, ADFI, and G:F (Hill et al., 2000; Case and Carlson, 2002; Carlson et al., 2004; Davis et al., 2004; Buff et al., 2005; Hollis et al., 2005; Yin et al., 2009; Martin et al., 2013), as well as reducing incidence of diarrhea (Hill et al., 2000; Ou et al., 2007; Wang et al., 2009; Pérez et al., 2011; Sales, 2013). On the contrary, several authors reported no or impaired growth performance when supplementing pharmacological levels of ZnO (Augspurger et al., 2004; Walk et al., 2013). It should be noted that the beneficial effect reported for pharmacological levels of ZnO only existed in the first 2 wk postweaning (Carlson et al., 2004; Buff et al., 2005) or 4 wk (Carlson et al., 1999; Yin et al., 2009; Shelton et al., 2011), which is consistent with the findings in the current study. Additionally, studies have also shown that the growth-promoting effect of ZnO could disappear when the inclusion level of ZnO in the diet is reduced to 500 mg/kg of Zn (Davis et al., 2004; Hollis et al., 2005). It has also been shown that the growth response provided by supplementation of pharmacological levels of ZnO in pigs weaned earlier than 15 d of age is greater than the response in pigs weaned after 20 d of age (Hill et al., 2001). These findings suggest that the beneficial effects of ZnO in weaning pigs are dose dependent and are influenced by weaning age.

Ever since the discovery of growth-promoting and diarrhea-reducing effects of ZnO in the 1990s (Poulsen, 1995), the mechanisms of action of ZnO that elicit these effects have been extensively investigated. Pharmacological levels of ZnO supplementation have been shown to maintain small intestinal microbiota stability (Katouli et al., 1999) and diversity (Vahjen et al., 2011), even though the percentage of *Escherichia coli* in ileal digesta (Li et al., 2001) and its viability (Roselli et al., 2003) were not affected. Additionally, it has been demonstrated that pharmacological levels of ZnO could reduce tight junction permeability, modulate cytokine gene expression (Roselli et al., 2003), reduce stem cell factor and mast cells in jejunum mucosa (Ou et al., 2007), improve redox status and prevent apoptosis of small intestine epithelial cells (Wang et al., 2009), as well as improving gut morphology (Li et al., 2001, 2006). Furthermore, pharmacological levels of ZnO could also exert the benefits via systemic routes, such as increasing serum insulin-like growth factor-1 (IGF-1) concentration (Li et al., 2006; Yin et al., 2009) and plasma ghrelin level (Yin et al., 2009), which may be responsible for the improved ADFI and ADG in the first 2 wk by ZnO supplementation in the current experiment. All these benefits could contribute to the improved gut health of weaning pigs, eventually leading to enhanced growth performance.

In the current study, Zn-MHAC supplementation tended to reduce ileum villus width in comparison to a pharmacological level of ZnO. It has been demonstrated that villus width is positively correlated with systemic inflammation in lactating cows (Kvidera et al., 2017), indicating that Zn-MHAC in the present study could lead to less gut and systemic inflammation, therefore leading to the improvement of G:F during days 14–28 compared with ZnO supplementation.

Due to increased attention on environmental pollution, supplementation of pharmacological levels of ZnO is not environmentally sustainable and an effective alternative for ZnO is needed. Chelated Zn, due to its stable structure, has been proven to be more bioavailable in both pigs and chicken compared with ZnO and Zn sulfate (Wedekind et al., 1994; Schell and Kornegay, 1996; Edwards and Baker, 1999). In our current study, we demonstrated that feeding 100 mg/kg Zn-MHAC yielded greater feed efficiency during phase 2 and similar growth performance in the entire nursery period as feeding 2,000 mg/kg Zn as ZnO in the first two phases and 100 mg/kg Zn as ZnO in phase 3, even though pharmacological levels of ZnO increased feed intake and growth rate in the first phase compared with Zn-MHAC. However, the definitive mechanism of this response is unclear. It is proposed that Zn-MHAC is more stable in the upper gastrointestinal tract, which could minimize the formation of Zn-phytate or Zn-Ca-phytate complex (Yi et al., 2007; Richards et al., 2010). It has been demonstrated that pharmacological levels of ZnO could reduce P digestibility and serum P concentrations in nursery pigs with or without phytase supplementation (Walk et al., 2013; Blavi et al., 2017). The improved P digestibility by Zn-MHAC supplementation compared with ZnO observed in the current study may partially explain the improved G:F in phase 2 and complementary growth in phases 2 and 3 in pigs fed Zn-MHAC compared with pigs fed a pharmacological level of ZnO.

### Cu Sources on Growth Performance and Gut Morphology

Although the NRC (2012) recommendation for Cu is 5–6 mg/kg for 5–25 kg nursery pigs, a high level of CuSO_4_ (150–250 mg/kg Cu) is widely used in weaning pigs to promote growth and improve feed efficiency (Cromwell et al., 1989; Jongbloed et al., 2011; Ma et al., 2015). In the past decades, other Cu sources have been heavily investigated in the weaning pigs. It has been demonstrated that Cu-proteinate was more effective than CuSO_4_ in terms of improving growth performance of nursery pigs (Veum et al., 2004), whereas Cu citrate yielded similar growth performance as CuSO_4_ in weaning pigs at the same inclusion level of Cu (Armstrong et al., 2004). In the current study, we demonstrated that Cu-MHAC supplementation led to greater ADG and feed to gain ratio in the entire nursery period compared with CuSO_4_ supplemented at the same levels of Cu, which was consistent with the findings from Zhao et al. (2014) and the meta-analysis results from Ma et al. (2015). These results indicated that Cu-MHAC may be more effective than CuSO_4_ in terms of promoting growth and enhancing feed efficiency.

Several theories have been proposed in terms of the mode of action of Cu on growth performance. One interesting study conducted by Shurson et al. (1990) led to the thought that the growth-promoting effect of high levels of Cu may be attributed to its antimicrobial property. However, it is not known whether this antimicrobial effect comes directly from Cu before its absorption or from Cu-containing bile acid after absorption and subsequent recycling back to the gut lumen. The second hypothesis is supported by Zhou et al. (1994a), which demonstrated that intravenous injection of Cu-histidinate could exert growth promoting effects. The antimicrobial effect of Cu is further supported by the evidence that Cu supplementation at 100 mg/kg as Cu-methionine or Cu-proteinate could increase the proportion of *Lactobacillus* while reducing the proportion of *E. coli* in the ileal digesta of broilers (Kim et al., 2011). It has also been demonstrated that supplementation of 30 mg/kg Cu from Cu-MHAC in broilers decreased the proportion of *Enterobacteriaceae* and *Fiumicutes* in the cecum digesta (Chen et al., 2016) compared with supplementation of 125 mg/kg Cu from CuSO_4_, which indicated that Cu-MHAC could modulate gut microbiota by shifting cecal microbiota to more beneficial microflora. Cu-MHAC supplementation may have a similar effect in the modulation of gut microbiota in the current study, which could also partially explain the increased growth rate and feed efficiency in the entire nursery period by Cu-MHAC supplementation compared with CuSO_4_.

The second theory in terms of the benefits of supplementing high Cu could be related to gut morphology improvement (Radecki et al., 1992; Hedemann et al., 2006; Zhao et al., 2007). In the current study, no difference was found between CuSO_4_ and Cu-MHAC treatments in terms of villus height and crypt depth in all small intestine segments of pigs at 42 d postweaning. It should be noted that in the previous studies with positive effect on gut morphology, high Cu supplementation is compared with no Cu supplementation. It is possible that such a positive effect may not exist when comparing two different Cu sources at the same Cu inclusion level. However, we cannot rule out the possibility that pigs fed diets containing Cu-MHAC had a lower turnover rate of small intestinal epithelial cells compared with those fed diets containing CuSO_4_. This hypothesis is evidenced by the fact that the turnover rate of epithelial cells in the upper small intestine was lower in pigs fed 250 mg/kg Cu as CuSO_4_ compared with no Cu supplementation, even when no difference was observed in gut morphology (Radecki et al., 1992).

The third potential mechanism in terms of growth-promoting or feed efficiency-enhancing effect of high Cu supplementation could be exerted via a systemic route. Evidence has demonstrated that increasing Cu supplementation in weaning pigs could increase serum mitogenic activity, liver superoxide dismutase activity, and pituitary growth hormone mRNA concentrations (Zhou et al., 1994a, 1994b) and elevate serum growth hormone and insulin-like growth factor-1, as well as enhance ghrelin mRNA expression in fundic region of stomach, and growth hormone-releasing hormone and suppressing somatostatin mRNA expression levels in hypothalamus (Yang et al., 2011, 2012; Wang et al., 2016). These anabolic factors could be the strong determinants for Cu to promote growth and enhance feed efficiency in pigs, which may also explain the improved growth rate and feed efficiency in the overall nursery period in pigs fed diets containing Cu-MHAC in the current experiment. Indeed, a recent study conducted by Gonzalez-Esquerra et al. (2019) demonstrated that nursery pigs fed Cu-MHAC at 50 mg/kg Cu led to increased serum growth hormone concentration than those fed CuSO_4_ at 160 mg/kg Cu.

The benefits of Cu-MHAC over CuSO_4_ may be derived from the increased bioavailability and stable structure in the gastrointestinal tract. It is demonstrated that Cu-MHAC had greater ATTD of Cu compared with CuSO_4_ when they are supplemented at the same Cu level (Liu et al., 2014). Compared with Cu-MHAC, CuSO_4_ easily dissociates and generates Cu ions in the stomach, which is a strong pro-oxidative substrate causing lipid oxidation and cell damage (Ajuwon et al., 2011). This may be the reason why plasma MDA concentration is lower in pigs fed Cu-MHAC compared with those fed CuSO_4_. The reduction of plasma MDA suggests better systemic antioxidant capacity and less oxidative stress, which may partially explain the improvement of growth performance by Cu-MHAC in the current study. The increased bioavailability and stability of the chelated structure of Cu-MHAC may be the initiating factor which potentiates the aforementioned mechanisms.

### Zn and Cu Sources and Their Interactions With Phytase on Growth Performance, Mineral Digestibility, and Metacarpal Mineral Concentrations

Chelated metals Zn-MHAC and Cu-MHAC, which are composed of one mole of Zn or Cu chelated with two moles of DL-2-hydroxy-4-(methylthio)butanoic acid (HMTBa) in coordinate covalent bonds, are stable in the upper gastrointestinal tract, which may minimize the formation of Zn-phytate and Cu-phytate complexes and allow more Zn and Cu to be absorbed by the epithelial cells in the jejunum and ileum (Yi et al., 2007; Richards et al., 2010). The reduction of Zn-phytate and Cu-phytate complexes could also improve P digestibility without phytase supplementation (Liu et al., 2014). In contrast, inorganic trace minerals, ZnO and CuSO_4_, are easily dissociated in the acidic pH of the stomach (Dintzis et al., 1995; Pang and Applegate, 2006); therefore, Zn-phytate, Cu-phytate, and Zn-Ca-phytate complexes are formed, rendering lower absorption of Ca, P, and these trace minerals (Leeson and Summers, 2001). Indeed, the current study demonstrated that ZnO supplementation reduced both Ca and P digestibility in the absence or presence of phytase compared with Zn-MHAC supplementation. The formation of Zn-phytate and Zn-Ca-phytate could also impair phytase efficacy as evidenced by the fact that ZnO supplementation led to 12.68% lower P digestibility compared with Zn-MHAC supplementation when phytase was supplemented at 500 FTU/kg in the diets.

It was interesting that phytase supplementation could improve G:F by 12% when Cu-MHAC was used as the Cu source in phase 2. However, the improvement of G:F by phytase supplementation was reduced to 5.26% when CuSO_4_ is used as the Cu source. It has been demonstrated that approximately 40–50% of Cu in CuSO_4_ is formed as insoluble Cu-phytate in the pH range 5.5–6.5, which restricted the efficacy of phytase to break down the phytate molecule to release P (Pang and Applegate, 2006). Indeed, increasing Cu levels as CuSO_4_ in the diets containing 600 FTU/kg resulted in a linear reduction of ADG and feed efficiency, as well as the apparent P retention, in broilers (Banks et al., 2004), which reinforces that CuSO_4_ at high-inclusion levels could impair phytase efficacy. In contrast, in the same in vitro model, the magnitude of inhibition on phytate hydrolysis by Cu lysine was much less compared with CuSO_4_ (Pang and Applegate, 2006), indicating that chelated Cu may be less soluble in the gastrointestinal tract and less Cu-phytate complex was formed. In the current study, however, Cu-MHAC did not affect P digestibility alone and did not affect phytase efficacy on P digestibility compared with CuSO_4_. Interestingly, it was demonstrated in the current study that the magnitudes of Ca, P, and ash deposition in metacarpals were greater by phytase supplementation when Cu-MHAC was used as the Cu source compared with CuSO_4_. This indicates that Cu-MHAC could improve Ca and P utilization in the body, which may partially explain the better feed efficacy by phytase supplementation when Cu-MHAC was used compared with CuSO_4_. The definite mechanism of improved Ca and P utilization by Cu-MHAC was not clear, which warrants further investigation.

In conclusion, ZnO supplementation at 2,000 mg/kg Zn was only effective in the first 2 wk postweaning, whereas Zn-MHAC supplementation at 100 mg/kg Zn could achieve better feed efficiency during phase 2 than pharmacological levels of ZnO, therefore, leading to no difference of growth performance in the entire nursery period. Low levels of Zn-MHAC may improve phytase efficacy on degrading phytate P compared to pharmacological levels of ZnO. Additionally, Cu-MHAC was demonstrated to be more effective compared to CuSO_4_ in terms of improving ADG and feed efficiency throughout the entire nursery period, which may be partially attributed to improved antioxidative status. Results indicate that Cu-MHAC and phytase may act synergistically to enhance feed efficiency and bone mineralization.
